# MedLexSp – a medical lexicon for Spanish medical natural language processing

**DOI:** 10.1186/s13326-022-00281-5

**Published:** 2023-02-02

**Authors:** Leonardo Campillos-Llanos

**Affiliations:** Instituto de Lengua, Literatura y Antropología (ILLA), CSIC (Spanish National Research Council), Albasanz 26-28, 28037 Madrid, Spain

**Keywords:** Medical Lexicon, Natural Language Processing, Word embeddings, Spanish

## Abstract

**Background:**

Medical lexicons enable the natural language processing (NLP) of health texts. Lexicons gather terms and concepts from thesauri and ontologies, and linguistic data for part-of-speech (PoS) tagging, lemmatization or natural language generation. To date, there is no such type of resource for Spanish.

**Construction and content:**

This article describes an unified medical lexicon for Medical Natural Language Processing in Spanish. MedLexSp includes terms and inflected word forms with PoS information and Unified Medical Language System$^{\circledR }$ (UMLS) semantic types, groups and Concept Unique Identifiers (CUIs). To create it, we used NLP techniques and domain corpora (e.g. MedlinePlus). We also collected terms from the *Dictionary of Medical Terms* from the Spanish Royal Academy of Medicine, the Medical Subject Headings (MeSH), the Systematized Nomenclature of Medicine - Clinical Terms (SNOMED-CT), the Medical Dictionary for Regulatory Activities Terminology (MedDRA), the International Classification of Diseases vs. 10, the Anatomical Therapeutic Chemical Classification, the National Cancer Institute (NCI) Dictionary, the Online Mendelian Inheritance in Man (OMIM) and OrphaData. Terms related to COVID-19 were assembled by applying a similarity-based approach with word embeddings trained on a large corpus. MedLexSp includes 100 887 lemmas, 302 543 inflected forms (conjugated verbs, and number/gender variants), and 42 958 UMLS CUIs. We report two use cases of MedLexSp. First, applying the lexicon to pre-annotate a corpus of 1200 texts related to clinical trials. Second, PoS tagging and lemmatizing texts about clinical cases. MedLexSp improved the scores for PoS tagging and lemmatization compared to the default Spacy and Stanza python libraries.

**Conclusions:**

The lexicon is distributed in a delimiter-separated value file; an XML file with the Lexical Markup Framework; a lemmatizer module for the Spacy and Stanza libraries; and complementary Lexical Record (LR) files. The embeddings and code to extract COVID-19 terms, and the Spacy and Stanza lemmatizers enriched with medical terms are provided in a public repository.

## Introduction

The demand for processing large volumes of health texts has triggered the need for domain resources combined with hybrid natural language processing (NLP) methods. Choosing the type of data or approach depends on aspects such as the task, the end-user (e.g. medical practitioners versus laymen) or the focus on precision versus recall. Transfer learning currently makes it possible to learn embedding representations or language models from massive data [1–4]. Nevertheless, two obstacles appear for transfer learning on health texts: 1) patient data are not available, since they require agreements with health institutions and anonymization; 2) texts need quality annotation with expert knowledge, which is time-consuming and labor intensive. These difficulties are more critical in languages for which less resources are available [5].

To alleviate this issue, research teams have resorted to unsupervised methods (i.e. without using labeled data by experts) [6] or to semi-supervised approaches (i.e. using a small amount of annotated data). A typical approach is pre-processing data with lexical/ontological resources, then train a machine-learning-based or deep-learning-based classifier. Former works have reported optimal results by means of pre-annotating medical texts [7, 8] or data augmentation using synonyms from a lexicon [9]. Recent teams have applied hybrid methods [10], integrating pre-annotation in the pipeline or using the prediction of a terminology-based system as features for a neural network model [11–13]. Thus, creating resources adapted to the medical terminology and health literature is beneficial to obtain optimal results [14].

In this context, we introduce MedLexSp, a computational medical lexicon for Spanish. Terms include linguistic information—lemmas, inflected forms and part-of-speech (PoS) tags—, Concept Unique Identifiers (CUIs) from the Unified Medical Language System$^{\circledR }$ (UMLS) [15], and UMLS semantic types and groups. MedLexSp is a dedicated lexicon that can be combined with complementary NLP methods. A use case is pre-annotating data for named entity recognition (NER). Although the tendency is to use domain gazetteers, a dedicated lexicon (with lemmas and PoS information) allows for developing enhanced annotation rules. MedLexSp can also feed general-purpose part-of-speech taggers of medical texts.

With regard to previous work [16], the latest version of MedLexSp (presented herein) has the following contributions:

- A broader coverage of medical terminology recorded in domain lexicons, the main contribution being aggregating terms from the *Dictionary of Medical Terms (DTM)* by the Spanish Royal Academy of Medicine [17].
- An updated list of term variants documented in real domain texts, namely the Spanish versions of MedlinePlus [18], and state-of-the-art annotated medical corpora: datasets used in recent shared tasks (CODIESP [19], CANTEMIST [20], PharmaCoNER [21]), the Chilean Waiting List Corpus (CLWC) [22] and the CT-EBM-SP corpus of texts about clinical trials [23].
- A richer representation of linguistic information: for each word form, the part-of-speech (PoS) tag and the following morphological data: gender, number, abbreviation/acronym (if applicable); and tense, person and mood for verbs.
- An experimental, unsupervised approach to gathering new terms by applying a semantic similarity measure and word embeddings trained on a text corpus about the COVID-19 pandemic.
- A standardized distribution format for lexical resources, the Lexical Markup Framework [24], which is an ISO standard.
- A lemmatizer module with MedLexSp forms and lemmas, to be used in downstream NLP tasks using the Spacy [25] and Stanza [26] python libraries.
- Complementary Lexical Record (LR) files with equivalences between acronyms and full forms, deverbal nouns and adjectives derived from nouns, and affixes.
- A demonstration of two use cases: firstly, applying the lexicon to pre-annotate a corpus of 1200 texts related to clinical trials. Secondly, part-of-speech tagging and lemmatizing 100 texts related to clinical cases. MedLexSp improved the scores for part-of-speech tagging and lemmatization compared to the default Spacy and Stanza python libraries.

The next sections provide an overview of similar resources, summarize the methodology to develop MedLexSp, describe the current stable version, and report the use cases (including the evaluation of part-of-speech tagging and lemmatization).

## Background

Health thesauri cluster terms and information about the type of term, semantic descriptors, concept identifiers and ontological relationships between them. Some resources aim at encoding clinical text—e.g. the Systematized Nomenclature of Medicine Clinical Terms (SNOMED-CT) [27]—and drug reactions—e.g. the World Health Organization Adverse Reactions Terminology (WHO ART) [28] and the Medical Dictionary for Regulatory Activities (MedDRA) [29]. Other controlled vocabularies such as the Medical Subject Headings (MeSH) [30] are used for indexing the scientific literature. Medical taxonomies have domain-specific knowledge—e.g. the Anatomical Therapeutic Chemical (ATC) drug classification system [31], the *Diagnostic and Statistical Manual of Mental Disorders vs. 5* (*DSM-5*$^{\circledR }$) [32] or the Online Mendelian Inheritance in Man (OMIM) catalog of genes and genetic disorders [33]. Some classifications are used for standardized codification: e.g. the International Classification of Diseases vs. 10 (ICD-10) [34] and the International Classification of Primary Care (ICPC) [35]. Lastly, medical lexicons [36–39] lack ontological relations, but organize terms and their linguistic information that can range from lemmas, word variants, and/or argument structure.

The Unified Medical Language System$^{\circledR }$ [15], supported by the National Library of Medicine, gathers together thesauri, ontologies and terminologies from 25 languages and 222 data sources. The previous-to-latest version (2022AA) contains over 4.5M concepts and more than 16.9M different concept names. Terms are encoded with a Concept Unique Identifier (CUI) and concepts are classed according to semantic types and groups [40]. For example, *chest* and *thorax* share the CUI (C0817096) and the semantic type Body Location or Region (ANAT group).

Medical lexicons enable the computational processing and actionable text mining of natural language texts. By incorporating the part-of-speech category, and gender, number and tense information of terms, lexicons are more powerful than standard gazetteers for basic tasks such as part-of-speech (PoS) tagging, lemmatization and natural language generation. If lexicons also include ontology data or codes from standard thesauri, synonym terms are clustered by means of concept identifiers.

When this is achieved, the interoperability across thesauri is easier and enhances concept normalization tasks.

**Fig. 1:**
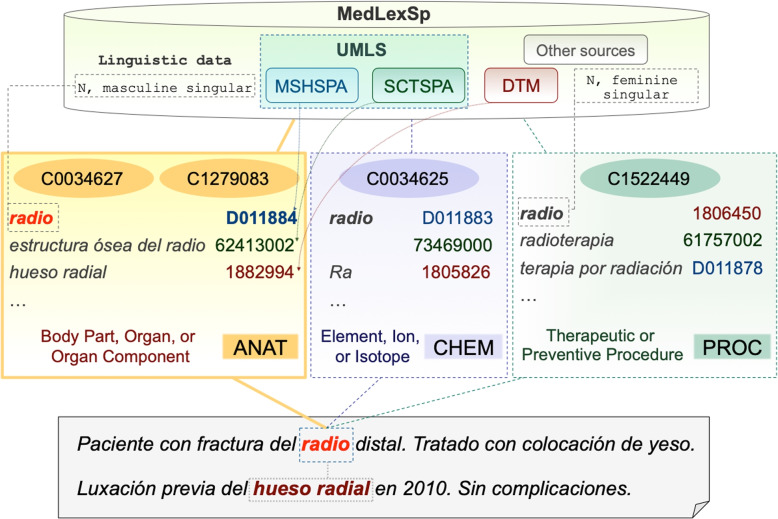
Example of UMLS-augmented lexicon to manage terminological variation (CUIs in ovals). Translation: ‘Patient with distal radius fracture. Treated with plaster cast. Previous dislocation of the radial bone in 2010. No complications.’

Figure 1 illustrates how an UMLS-augmented medical lexicon can manage terminological variation. The term *radio* is ambiguous in Spanish: it can refer to the arm bone (‘radius’), the chemical element (‘radium’) and it can also be an abbreviation, standing for ‘radiotherapy’ or ‘radiograph’. Each concept has one or more CUIs and a different UMLS semantic type and group; respectively: C0034627 and C1279083, ANAT (Body Part, Organ, or Organ Component); C0034625, CHEM (Element, Ion, or Isotope); and C1522449 (‘radiotherapy’) (Therapeutic and Preventive Procedure, PROC), C1306645 or C1306645 (‘radiograph’) (Diagnostic Procedure, PROC). Each terminology and ontology source in the UMLS provides variant forms, and a CUI clusters the corresponding synonyms. For example, the same CUI (C0034625, for the chemical element) is used for the term *radio* (‘radium‘), as registered in the MeSH thesaurus (code: D011883) and SNOMED-CT (code: 73469000). In MedLexSp, UMLS CUIs were also added to terms from other sources such as the *Dictionary of Medical Terms* (e.g. the abbreviation *Ra*). In the sentence *Paciente con fractura del radio distal* (‘Patient with distal radius fracture’), *radio* refers to the body part, and it is a masculine singular noun. Note that when *radio* is the abbreviation of ‘radiotherapy’ or ‘radiograph’, it is a feminine singular noun; and if *radio* refers to the chemical element, it only appears in the singular form. This linguistic information can be used for disambiguation, in combination with cues from the linguistic context. In the case that a co-reference item occurs in the same text (e.g. *hueso radial*, ‘radial bone’), variant terms can be mapped to the same concept code (C0034627 and C1279083 in the UMLS).

In contrast to other languages such as French or English, a unifying comprehensive lexicon does not exist for the Spanish language to date. There is not a resource similar to the Specialist Lexicon [36], the Biolexicon [38] or the Unified Medical Language for French [37]. Although different teams have made dispersed efforts to build a Spanish MetaMap [41, 42], these initiatives, as far as we know, did not achieve a Spanish medical lexicon for NLP. This situation is unfortunate, given that Spanish is one of the most spoken languages worldwide (with 548 million speakers in 2022, according to the Ethnologue [43]).

## Construction and content

**Fig. 2:**
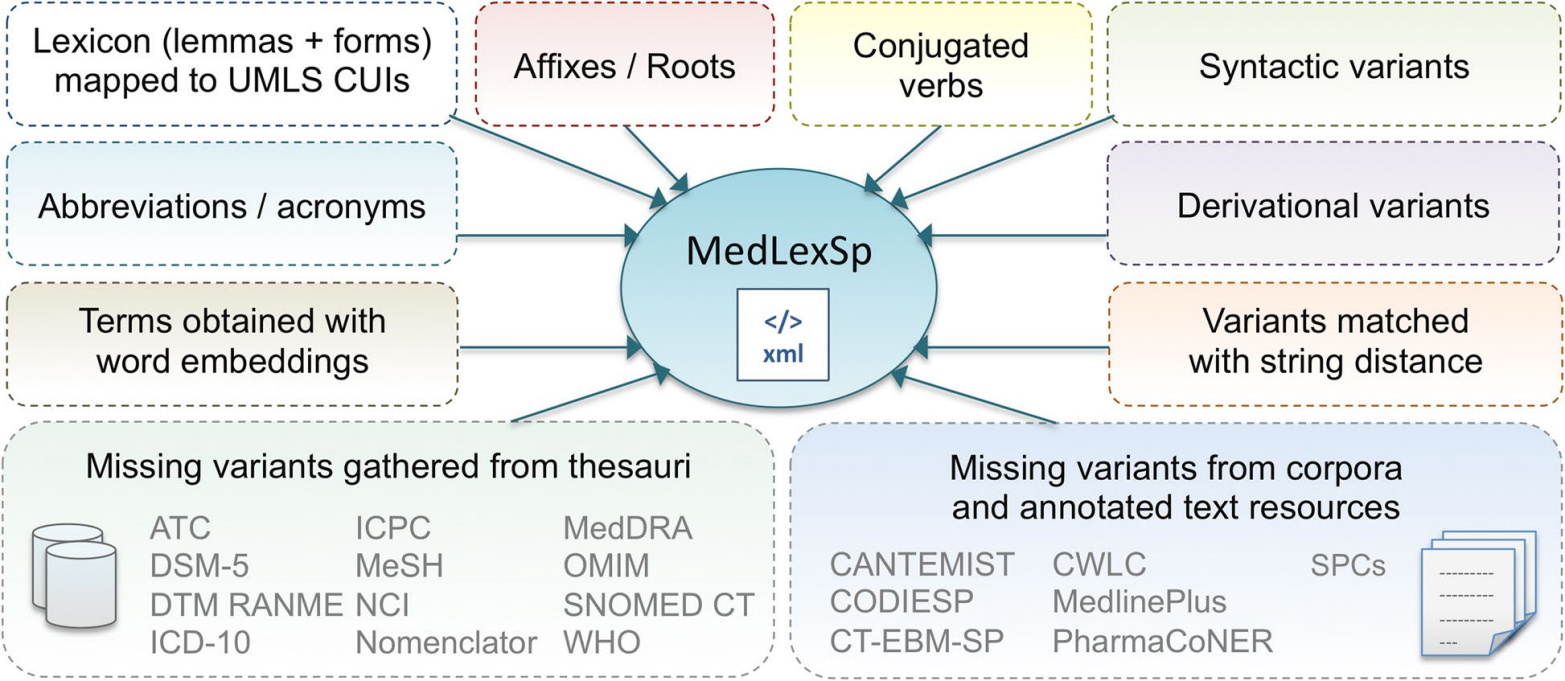
Methods applied to collect the MedLexSp lexicon

This section summarizes the methodology reported in [16], and explains the word-embedding-based method to collect new terms about the COVID-19 pandemic. Figure 2 depicts the approaches to create MedLexSp. Note that methods might be generalized across languages provided that similar resources are available.

### Base list

First, we used a list of medical terms developed by [44]—hereafter, the *base list*. This resource was collected from a corpus of Spanish medical texts (around 4 million tokens) by applying rules, part-of-speech tagging and medical affixes, comparing general and domain corpora, and statistical methods. The *base list* amounted to 38 354 tokens (base and variant forms). Not all the terms in the list were used to prepare MedLexSp. This lexicon was aimed at concept normalization, mainly using standard terminologies. To do so, we used the UMLS, thus MedLexSp only includes terms mapped to Concept Unique Identifiers (CUIs). Approximately, 47.61% entries of the original *base list* were mapped to CUIs, applying an exact match criterion. For example, the CUI for *neoplasia* (‘neoplasm’, C0027651) was not assigned to *neoplasia benigna* (‘benign neoplasm’, C0086692), because these terms refer to different concepts. Once a stable list of terms was achieved, MedLexSp was enriched with several sources, as explained in the following sections.

### Acronyms and abbreviations

We reused a dictionary collected by medical doctors [45], acronyms from Wikipedia, and the resources provided in the Biomedical Abbreviation Recognition and Resolution Challenge [46]. Acronyms and abbreviations were matched to UMLS CUIs semi-automatically and revised manually. This revision was essential because many are ambiguous: e.g. *IM* stands for *insuficiencia mitral* (‘mitral insufficiency’), *infarto de miocardio* (‘myocardial infarction’) or *intramuscular* (‘intramuscular’). Other items are invariant in English and Spanish (e.g. *kg*, ‘kilogram’) and the mapping was automatic. With these methods, the CUI of each acronym (e.g. *EV*, C0014383) was assigned to each full form (*enterovirus*), and vice versa. A complimentary list of equivalences between acronyms/abbreviations and full forms (LR_abr.dsv) is also distributed.

### Affixes and roots

We translated items from the Specialist Lexicon [36] (e.g. *reno-*, ‘kidney’), and reused a list from a previous work [47]. This lists gathers suffixes recommended by the World Health Organization [48] to coin new drug terms: e.g. *-cilina* (‘-cilin’) is used for a penicillin drugs. Morphological variants of affixes were created, including gender/number alternations (e.g. *-scópico*, *-scópica* and *-scópicos*, ‘-scopic’) or variants with tilde (*-scopia* and *-scopía*, ‘-scopy’). Then, a subset of items were mapped to UMLS CUIs, and variants were clustered for each base form and CUI. For example, the suffix *-cilina* was mapped to CUI C0030842 for ‘penicillins’, and all form variants (*-cilina, -cilinas*) were clustered. A complimentary Lexical Record file (LR_affix.dsv) provides the equivalence between affixes/roots and their meanings.

### Conjugated verbs

Medical events are commonly expressed with nouns (*sangrado*, ‘bleed’), but verbs may be used as well (*sangrar*, ‘to bleed’, C0019080). For this reason, state-of-the-art lexicons [14, 36, 49] gather verb terms, and we proceed similarly in MedLexSp. From a list of medical verbs, we generated conjugated variants by using a python script and the lexicon of a Spanish part-of-speech tagger [50]: e.g. *sangrar* (‘to bleed’) $\rightarrow$*sangra* (‘he/she/it bleeds’), *sangrando* (‘bleeding’), *sangrado* (‘bled’)... Then, the CUI of each noun term was assigned to the corresponding verb term.

### Derivational variants

By using lists of morphological and semantic variants, we mapped noun terms to adjective variant forms: e.g. *hígado*, ‘liver’ $\leftrightarrow$*hepático*, ‘hepatic’ (C0023884). We also matched deverbal nouns and verbs (*diálisis*, ‘dialysis’ $\leftrightarrow$*dializar*, ‘to dialyze’, C4551529). Note that larger lists were collected, but only a subset (801 items) was mapped to UMLS CUIs. The full lists of deverbal nouns are also released as complementary lexical record (LR) files. The list of deverbal nouns (LR_n_v.dsv) amounts up to 535 entries. The list of adjectives derived from nouns (LR_adj_n.dsv) gathers 2366 entries, including morphological variants (e.g. *abdomen*$\leftrightarrow$*abdominal*) and non-morphologically related pairs (e.g. *oncológico*, ‘oncological’ $\leftrightarrow$*cáncer*, ‘cancer’).

### String distance metrics

We computed string distance metrics [51] of $\le$2 between the terms with a CUI available, and unattested variants in thesauri. The selected candidates were revised manually, to match CUIs to new variant forms. This procedure was useful for character-level variants (e.g. *viriasis*$\leftrightarrow$*viriosis*, ‘viral infection’, C0042769), hyphenated variants and tokenization variants (*betabloqueante*$\leftrightarrow$*beta-bloqueante*$\leftrightarrow$*beta bloqueante*, ‘beta-blocker’, C0001645).

### Syntactic variants of terms

We created variants of multi-word terms in available thesauri. Word order was swapped, and the UMLS Concept Unique Identifier of the original form was assigned to the new variants. The form variants were obtained automatically with a python script, and then they were revised manually. With this method, for example, the CUI of *virus respiratorio sincitial* (‘respiratory syncytial virus’, C0035236) was matched to the variant form *virus sincitial respiratorio* (‘respiratory syncytial virus’).

### Terms from thesauri, dictionaries and knowledge bases

Health thesauri, knowledge bases, classifications and taxonomies were used to widen the coverage of terms. We collected variants of terms in the *base list* by means of UMLS CUIs mapped to alternative forms from the following resources:

1. The Anatomical Therapeutic Chemical (ATC) Classification [31]: this is a WHO standard to classify medical drugs according to their therapeutic and pharmacological properties. It comprises five levels, from the system or organ class (e.g. nervous system drugs) to the active ingredient (e.g. diazepam). By including data from the ATC, MedLexSp ensures to provide a exhaustive range of medical drug terms.
2. The *Dictionary of Medical Terms (DTM)* by the Spanish Royal Academy of Medicine [17]: this is the key contribution of this version of MedLexSp. This resource covers both technical words and consumer health terms. Note that the *DTM* also records frequent misspelled terms (e.g. *kinasa instead of *cinasa*, ‘kinase’), and MedLexSp also includes some of these misspelled forms. From 40 076 concept entries, we included 30 733 entries (76.7%) that were mapped to UMLS CUIs automatically or manually.
3. The International Classification of Diseases vs. 10 (ICD-10) [34]: the WHO maintains this standard terminology and classification system, which is available in 40 languages for clinical diagnose and epidemiology. Terms are grouped in subdomains according to the system/organ class (e.g. respiratory system disorders), and the 10th version is currently the most implemented. A subset of terms from the International Classification of Diseases for Oncology (ICD-O-3) was also collected. Terms from both classifications enable an extensive coverage of standard disease-related terms.
4. The International Classification of Primary Care (ICPC) [35]: this is a taxonomy of terms, ranged in 17 chapters related to disorders according to body systems (e.g. digestive, circulatory or neurological conditions, among others). This resource ensures a wide coverage of terms related to primary care.
5. The Spanish version of the *Diagnostic and Statistical Manual of Mental Disorders, 5th ed* (*DSM-5*$^{\circledR }$) [32]: terms were mapped from the English codes in the UMLS using CUIs. This subset of terms in the lexicon covers an adequate range of mental disorders and psychiatric conditions.
6. The Medical Dictionary for Regulatory Activities (MedDRA) [29]: this classification and coding system is aimed at pharmacovigilance. The domain of MedDRA includes signs and symptoms, disorders and diagnostics, tests, labs and procedures, and social or medical history. It is available in 14 languages, and the Spanish translation was used. Thus, MedLexSp includes terms for most adverse events of pharmaceutical drugs. The subset of terms from MedDRA cannot be not distributed publicly owing to use restrictions.
7. The Medical Subject Headings (MeSH) [30]: the National Library of Medicine (NLM) maintains and updates this thesaurus with the purpose of indexing and classifying the biomedical literature. Available in several languages, the BIREME is responsible for the Spanish translation. Term classes range from anatomy and diseases to chemicals and drugs or analytical, diagnostic and therapeutic techniques, among others. This guarantees a wide coverage of medical subdomains using a terminological standard. MeSH terms were incorporated by means of a license agreement with BIREME.
8. The National Cancer Institute (NCI) Dictionary [52]: this is a comprehensive glossary of cancer-related terms (cancer types, therapeutic and diagnostic procedures, or chemotherapeutic drugs). There is a consumer-oriented version available online, so both technical and laymen terms were included.
9. OrphaData [53]: the Orphanet Rare Diseases Ontology (ORDO) is a controlled vocabulary and ontology for rare diseases, and a list of rare disorders mapped to reference terminologies. An XML file is available in several languages, including Spanish, and these data were processed to extract lists of terms and codes. We provide a companion script to extract the data (it could also be used for other languages: e.g. English, French, Italian or Portuguese). This resource provides an extensive coverage of rare diseases.
10. The Spanish Drug Effect database (SDEdb) [54]: this resource gathers terms related to adverse effects obtained from drug packages and medical web sites and social media. This database provides both new drug-related terms and laymen variants of technical words (e.g. *deprimido*, ‘depressed’, is more frequently used in consumer social media than *depresión*, ‘depression’).
11. The Nomenclator [55]: this is a rich database of drug brand names, generic compounds and international non-proprietary medication names prescribed in Spain. Data are available in several file formats, even an XML file.
12. The Systematized Nomenclature of Medicine Clinical Terms (SNOMED-CT) [27]: a comprehensive nomenclature and ontology covering medical findings, procedures, body structures, pharmaceutical products and qualifiers. The College of American Pathologists developed it initially, and is currently supported by the International Health Terminology Standards Development Organisation (IHTSDO). It is one of the largest resources and the main clinical terminology for clinical coding worldwide. Because this is a resource with use restrictions, the subset of terms from SNOMED-CT is not shared.
13. The Online Mendelian Inheritance on Man (OMIM) [56]: the John Hopkins University maintains this large catalog of genes and genetic diseases resource. We mapped OMIM data from English terms in the UMLS (using CUIs) and codes from OrphaData. Since OMIM combines genetic data and descriptions of genetic disorders, the fact of including OMIM terms enriches MedLexSp with these types of information.
14. The WHO Adverse Drug Reactions (WHO-ART) terminology [28]: this dictionary was compiled for pharmacovigilance and is available in several languages. We used the Spanish translation in MedLexSp to include more than 2800 terms related to adverse events.

### Terms from domain corpora

First, we extracted terms from 306 Summaries of Product Characteristics (SPCs) in the EasyDLP corpus [57], and from the Spanish versions of MedlinePlus [18] (for consumer health terms of disorders and lab tests). Using these corpora, most drug names and pharmacological substances are represented in MedLexSp.

Second, we used a domain corpus (+4M tokens) [58] to compute frequencies of the terms from MeSH and SNOMED-CT. Because these thesauri are too large, this strategy was applied to add a subset of terms that could be revised in a reasonable time and manner. Namely, a total of 48 188 term entries from SNOMED-CT were revised, and 20 649 term entries from MeSH.

Third, we added missing entities that were annotated in recent medical corpora; some of these resources have being used in competitions or shared tasks:

1. The Pharmacological Substances, Compounds and proteins Named Entity Recognition (PharmaCoNER) corpus [21]: this dataset gathers 1000 texts annotated with drug entities and proteins, which were normalized to SNOMED-CT [27] codes. Adding these entities to MedLexSp ensures a large coverage of terms related to pharmacological and biochemical substances.
2. The Clinical Case Coding in Spanish (CODIESP) corpus [19]: 1000 clinical cases published in scientific literature that were employed in a shared task for coding disorders using the International Classification of Diseases vs. 10 (ICD-10). By incorporating terms from this dataset, most disorders and conditions considered in the ICD-10 classification were added to MedLexSp.
3. The CANcer TExt Mining Shared Task (CANTEMIST) corpus [20]: 3000 annotated clinical cases about cancer used in a shared task for named entity recognition, normalization and coding of tumor morphology and codes of the International Classification of Oncology Diseases (ICD-O). With this dataset, MedLexSp provides a large typology and coverage of oncological diseases.
4. The Chilean Waiting List Corpus [22]: a collection of medical referrals annotated with semantic entities ranging from disorders, findings, drugs or procedures. The first version of the corpus was used (900 referrals).
5. The Clinical Trials for Evidence-based Medicine in Spanish (CT-EBM-SP) corpus [23]: this is a collection of 1200 texts related to clinical trial studies published in journals from the SciELO repository [59] and clinical trial announcements from EudraCT [60]. This dataset was employed as use case, where MedLexSp was applied to pre-annotate the data with UMLS semantic groups from the health domain, before manual revision (see Use cases section). The CT-EBM-SP resource is normalized to UMLS CUIs, so the inclusion of variant terms into the lexicon was easier. With this corpus, terms related to experimental drugs, interventions and clinical trial methods are represented in MedLexSp.

For the selected terms, we added UMLS CUIs, semantic types and groups, and PoS and morphological data (see Acquiring morphological data of terms section).

### Combining a similarity measure and word embeddings

To incorporate new terms related to the COVID-19 pandemic, we tested a complementary approach to state-of-the-art rule-based techniques [61]. We employed a similar method to that applied for terminology expansion using patient blogs and electronic health records [62–64]. The experiment was based on: 1) A set of 20 seed words related to the COVID-19 pandemic; 2) An unsupervised approach combining a word embedding model and a similarity metric (the cosine value) to obtain semantically similar new words; 3) A collection of texts (+6M tokens) about the COVID-19 pandemics; and 4) A word embedding model trained on a collection of texts related to the pandemic topic. With this method, the coverage of MedLexSp was expanded with terms not available in the lexicon, but evidenced in a corpus.

As *seed words*, we used the following terms related to COVID-19: *arbidol, camrelizumab, COVID-19, coronavirus, confinamiento* (‘lockdown’), *cuarentena* (‘quarantine’), *colchicina* (‘colchicine’), *danoprevir, EPI* (‘Individual Protection Equipment’), *EPP* (‘Personal Protective Equipment’), *hidroxicloroquina* (‘hydroxychloroquine’), *favipiravir, FFP2, leronlimab, N95, opaganib, remdesivir, SARS-CoV-2, umifenovir*, and *Wuhan*. These terms were selected from COVID-19 glossaries available online [65], or appeared frequently in news media or scientific publications.

The unsupervised approach used the nearest neighbors algorithm by computing semantic similarity values. This similarity was measured by obtaining the word vectors of each *seed term* and token in several word embedding models, and calculating the cosine similarity (CS) value between vectors:

$$\begin{aligned} similarity = \cos ({ \vec{s} },{ \vec{w}})= \frac{{ \vec{s} } \cdot { \vec{w} } }{ \Vert { \vec{s} }\Vert \cdot \Vert { \vec{w} } \Vert } = \frac{ \sum _{i=1}^{n}{{ \vec{s} }_i \cdot { \vec{w} }_i} }{ \sqrt{\sum _{i=1}^{n}{({ \vec{s} }_i)^2}} \cdot \sqrt{\sum _{i=1}^{n}{({ \vec{w} }_i)^2}} } \end{aligned}$$

where $\vec{s}$ is the vector of the *seed term* and $\vec{w}$ is the vector of a *word* in an embedding model. A cosine similarity of 1 indicates that token and term are identical, whereas a value of 0 means that the vectors are completely dissimilar—and, consequently, their meanings. The 50 candidate words with the highest CS values were retrieved for each term. The following is an example for the seed term *remdesivir* (only showing the first 10 nearest neighbors):

- remdesevir     0.8997
- veklury     0.7677
- veklury®     0.7516
- antiviral     0.72
- acalabrutinib     0.7145
- oseltamivir     0.7143
- baricitinib     0.6989
- darunavir     0.6949
- tofacitinib     0.693
- fármaco     0.6855 (‘medical drug’)

The example shows that the nearest neighbors are spelling variants (*remdesevir*), the brand name of the drug (*veklury®*), the name of the drug class (*antiviral*) or other antiviral agents (*oseltamivir, darunavir*). Note that a depth of 10 nearest neighbors was also tested, but the coverage of new terms was not satisfactory, since most of the 10 nearest neighbors obtained were misspellings or tokenization errors. The procedure involved looking up each out-of-vocabulary nearest neighbor—i. e. tokens not recorded in MedLexSp—by means of a python script, and checking manually whether the candidate new words were registered in the UMLS.

The word embedding models used to compute the word vectors were tested according to the different hyperparameters and configurations that yielded better results in terms of recall. First, we tested already-available word embedding models, namely the Spanish Biomedical and Clinical Word Embeddings in fastText [66]. These were trained on a large corpus exceeding 900M tokens, covering resources such as Wikipedia, the SciELO text corpus, texts from EMEA and the Spanish Register of Clinical Trials (REEC), and also a small proportion of COVID-19 clinical cases. We applied different pretrained model variants of 10, 100 and 300 dimensions (cased and uncased), and both architectures featured in fastText [2] (SkipGram and CBOW).

Despite the large volume of data used to train those embeddings, the quality of the nearest neighbors gathered was not satisfactory. Different studies have previously shown that a larger volume of data does not always yield the best results [67–69]. For example, the authors of [67] compared systematically general and domain-specific word embeddings for clinical and biomedical information extraction tasks. They did not found a correlation in performance between general and medical or clinical embeddings. Nevertheless, they did observe that word embeddings trained on text sources from local, smaller corpora yielded better results for local or *ad hoc* tasks. Likewise, the authors of [68] compared fastText and ELMo embeddings [3] trained on general domain texts and on specialized data for text classification and natural language understanding tasks. Their results were less conclusive: embeddings trained on a larger general corpora only yielded higher scores in the text classification task; but in the NLU task, the best results were obtained with embeddings trained with smaller data (but domain-specific, i.e. electronic health records). Another research team [69] compared public available pretrained language models and word vectors for a named entity recognition task (they used several biomedical and general datasets). Their outcomes tend to support that word vectors and language models trained on smaller sources (but with similar content and vocabulary to the target task) achieve comparable or higher scores than models trained on larger sources. The impact of corpus size or general versus domain-specific training texts is an aspect that needs further research.

Our approach for this task followed the assumption that models trained on smaller corpora, with texts more related to our task, would perform better. This is the reason why Spanish texts related to the COVID-19 pandemic were crawled from the Web to train word embeddings. Crawled web sites correspond to repositories of scientific or medical articles (Cochrane, PubMed) or health and research institutions (public information available in the Spanish National Research Council, the Spanish Ministry of Health, several regional health administrations, and in different National Institutes of Health (NIH), such as the National Cancer Institute). Other crawled sites were government drug agencies such as the Spanish Agency of Medicines and Medical Devices, the European Medicines Agency or the Food and Drug Administration. Information from independent agencies or journals was also crawled (e.g. Agencia SINC, The Conversation) in addition to data from Wikipedia. A list of text sources is provided in the companion GitHub repository. To select the sites, we searched on the Internet for COVID-related words and crawled sites ensuring quality content and created or supported by scientists or health experts. For PubMed, we used the following query: ((Spanish[Language]) AND (COVID-19[Title/Abstract])) AND (SARS-CoV-2[Title/Abstract]). The text collection exceeds 6M tokens, but we cannot redistribute it because some content is copyrighted. However, we release the trained embeddings and the source code to replicate our experiments.

Before training the models, texts were normalized (e.g. urls or non-utf-8 characters were removed) and white spaces were inserted between each token and punctuation sign (e.g. commas or dots). We used fastText [2] to train vectors of dimension 100 with SkipGram, and experimented with minimum term frequencies of 3 and 5.

### Results of the semantic similarity approach using word embeddings

With this method, we gathered a total of 222 term entries (491 form variants corresponding to 158 unique CUIs). The best results were obtained with the word embeddings trained on COVID-19-related texts using the SkipGram configuration, 100 dimensions, a minimum token frequency of 3 and a window size of 5. Note that the recall of out-of-vocabulary items was rather large. Table 1 shows that the number of out-of-vocabulary (OOV) items was around 70% of the total nearest neighbors obtained (1000 items: 50 nearest neighbors for each of the 20 seed words). With the word embeddings trained on COVID-19 texts, OOVs ranged from 67.7% (model trained with minimum token frequency of 5) to 69.0% (model trained with minimum frequency of 3). However, most of the OOVs were spelling errors (e.g. *covd-19*), tokenization mistakes or words with hashtags (e.g. #*virus*). Many OOVs were ATC codes for drugs, pharmaceutical brand names, acronyms of health organizations and emojis (given that many texts come from the web). Only a small subset of OOVs were found in the UMLS and were assigned a CUI. With the best model configuration, a 11.3% of the OOVs could be matched to UMLS CUIs.

**Table 1:** Results of the nearest neighbors (NN) experiments with different word embedding models

Word embedding model	OOVs	% of NN	OOVs mapped to CUI	% of OOVs mapped to CUI
SBCWE, uncased,	740	74.0%	48	6.49%
SkipGram, d=100				
SBCWE, uncased,	742	74.2%	51	6.88%
CBOW, d=100				
SBCWE, uncased,	762	76.2%	45	5.91%
SkipGram, d=50				
SBCWE, uncased,	732	73.2%	47	6.42%
CBOW, d=50				
SBCWE, uncased,	752	75.2%	46	6.12%
SkipGram, d=300				
SBCWE, uncased,	741	74.1%	50	6.75%
CBOW, d=300				
COVID-19 corpus, uncased,	677	67.7%	56	8.27%
SkipGram, d=100, min=5				
COVID-19 corpus, uncased,	690	69.0%	78	**11.30%**
SkipGram, d=100, min=3				

Abbreviations: *CUI *UMLS concept unique identifier; *d*: embedding dimensions;

*NN *Nearest neighbors, *OOVs *Out-of-vocabulary items;

*SBCWE *Spanish Biomedical and Clinical Word Embeddings

**Fig. 3:**
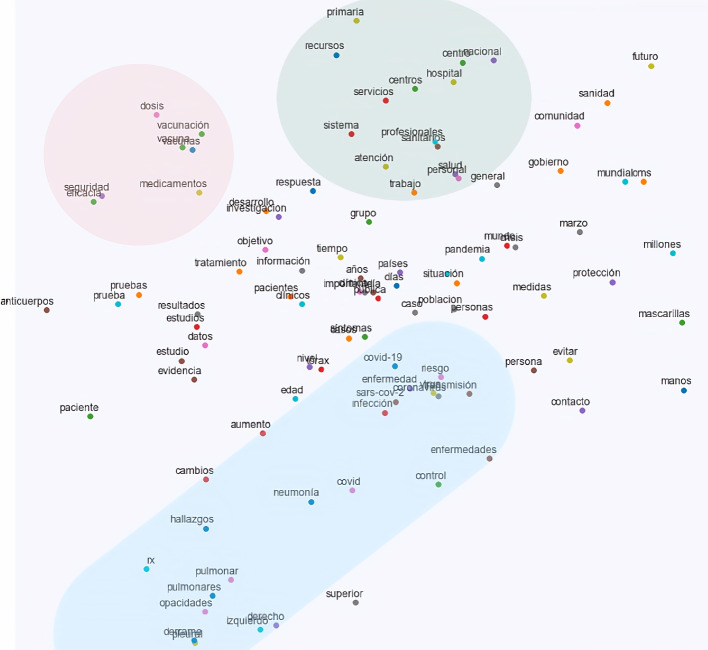
t-SNE visualization of the 100 most frequent words in the corpus

As a qualitative analysis of the word embeddings used in the experiments, Fig. 3 shows the t-Distributed Stochastic Neighbor Embedding (t-SNE hereafter) [70] projection of the 100 most frequent words in the corpus. For this figure, we used a SkipGram word embedding model of 100 dimensions (minimum corpus frequency of 5). Stopwords (e.g. prepositions and articles) are not shown. In this figure, specific words related to findings, pathological conditions or body locations tend to appear in the middle to lower left region (marked in the blue area; e.g. *infección*, ‘infection’; *COVID*; *neumonía*, ‘pneumonia’; *pulmonar*, ‘pulmonary’; *opacidades*, ‘opacities’). Words related to drugs or procedures (e.g. *vacunación*, ‘vaccination’; *vacuna*, ‘vaccine’; *dosis*, ‘dosage’; *medicamentos*, ‘drugs’) are shown in the upper left region (marked in the red area). Lastly, words related to medical institutions, professionals or general care tend to occur in the upper region (marked in the green area; e.g. *hospital*, ‘hospital’; *sanidad*, ‘healthcare’; *profesionales*, ‘professionals’). Even though this is a shallow analysis (and only considers mono-word items), it shows that this unsupervised method can cluster words in semantically similar classes according to their position in the vector space.

**Fig. 4:**
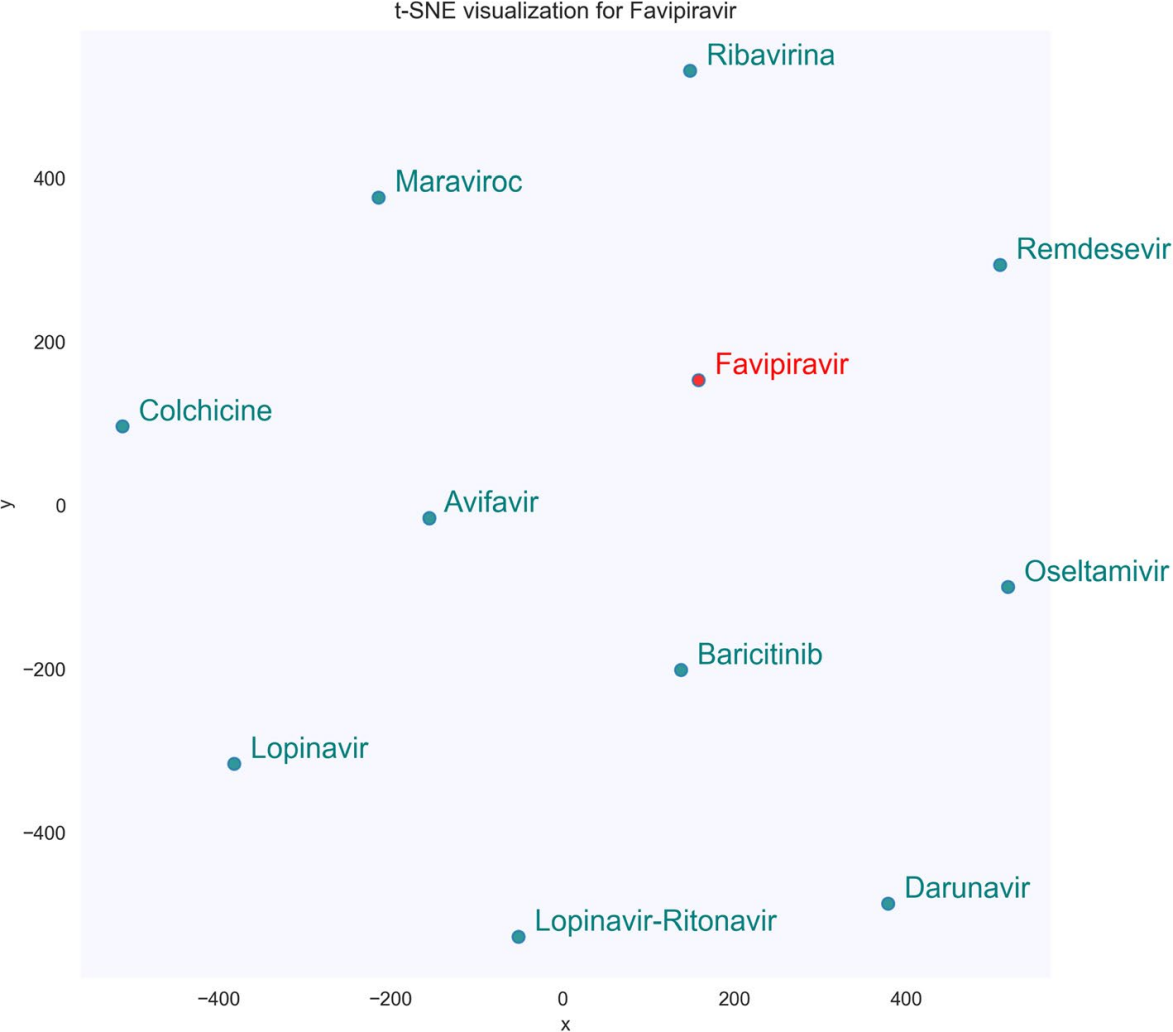
t-SNE visualization of the 10 most similar terms of the seed term *fapiravir*

**Fig. 5:**
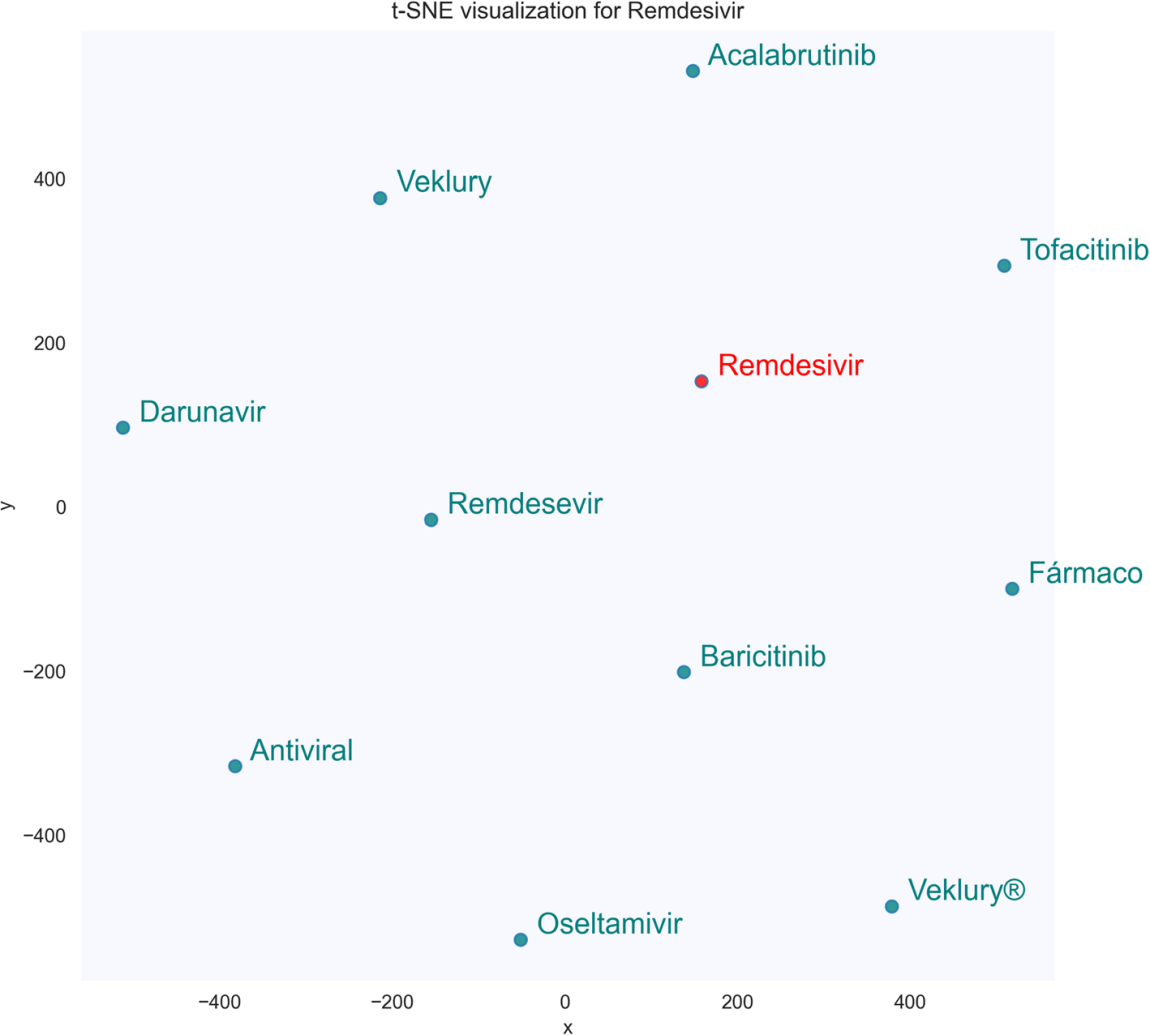
t-SNE visualization of the 10 most similar terms of the seed term *remdesivir*

These data can be more clearly displayed in Figs. 4 and 5, which show the t-SNE visualization of the 10 most similar terms for the seed terms *remdesivir* and *favipiravir*, two antiviral agents that were tested to treat the COVID-19 infection. For this figures, the word embedding model used also features 100 dimensions and a minimum term frequency of 5 (SkipGram configuration).

### Acquiring morphological data of terms

After collecting the terms and variants with the methods explained, the last stage involved enriching the lexicon with linguistic information. This morphological information can be used in NLP tasks such as part-of-speech tagging, lemmatization or natural language generation of medical texts. In addition to adding these types of data to mono-word terms, multi-word terms were also considered. In a similar manner to the Specialist Lexicon, multi-word terms were labeled with the category of the head word: e.g. *enfermedad de Lyme* (‘Lyme disease’) has label *N* (noun). Different approaches were applied to enrich the lexicon with the part-of-speech category of terms and morphological data of each variant form:

1. Terms registered in the *Dictionary of medical terms* [17] record the category and morphological data such as gender and number of noun or adjectives. Therefore, this type of information was leveraged in MedLexSp.
2. The subset of terms included in DELAS electronic dictionaries for Spanish [71] was collected along with the linguistic information there encoded.
3. The subset of medical terms encoded in the lexicon of the SPACCC PoS tagger [72] was processed and their linguistic information was added to MedLexSp.
4. The GRAMPAL tagger [50] was applied to predict the part-of-speech of mono-word terms for which no morphological data were obtained using the previous methods.
5. Lastly, with regard to multi-words, the procedure was to leverage the information of the head word, given that the head determines the analysis of the constituent. For example, *síndrome de Asperger* (‘Asperger syndrome’) is labeled as noun masculine singular (the same as *síndrome*). Thus, a script pre-processed the head word of each multi-word, and assigned the part-of-speech and gender/number of the head to the full entity. The list obtained with this approach was corrected manually.

All the PoS information and morphological data of terms and form variants were revised.

### Descriptive Statistics

We report in Table 2 the count of entries in MedLexSp according to each method to assign UMLS CUIs, and the number of concept codes from each data source. Note that the full count exceeds the number of term entries because some were extracted using different approaches in parallel. Table 3 shows the counts of lemmas, word forms, and CUIs, along with the number of PoS categories. Most entries are nouns, adjectives or proper names (e.g. drug brand names: *apocard*$^{\circledR }$).

**Table 2:** Count of entries according to each method, and count of concept codes from each source

Method	# entries
1. Abbreviations / acronyms	6679
2. Affixes / roots	914
3. Conjugated verbs	867
4. Derivational variants	801
5. String distance method	1463
6. Syntactic variants	134
7. Terms collected using word embeddings	222
8. Terms from corpora:	
CANTEMIST	2619
CODIESP	3384
CWLC	1511
MedlinePlus	1682
PharmaCoNER	173
SPCs (EasyDLP corpus)	837
9. Thesauri, dictionaries and knowledge bases:	**# codes**
DTM	30 816
ATC + Nomenclátor + SDEdb	2931
DSM-5	188
ICD-10	19 888
ICPC	179
MedDRA	20 209
MeSH	20 911
NCI	7621
OMIM	15 143
OrphaData	10 741
SNOMED-CT	53 893
WHO	2811
Other	4939

**Table 3:** Descriptive statistics of the lexicon and count of part-of-speech categories

	**Lemmas**	**Forms**	**CUIs**
**Single-words**	33 988	130 915	-
**Multi-words**	66 899	171 628	-
**Total**	100 887	302 543	42 958
**M per CUI**	2.35	7.04	-
**SD**	2.16	15.43	-
**Max / Min**	30 / 1	475 / 1	-
**PoS**	**Example**		**Count (%)**
**N**	*hígado* (‘liver’)		90 188 (89.40)
**ADJ**	*hepático* (‘hepatic’)		4933 (4.89)
**NPR**	*Streptococcus*		2786 (2.76)
**ADJ/N**	*neonato* (‘newborn’)		1033 (1.02)
**AFF**	*reno-* (‘kidney’)		913 (0.90)
**V**	*sangrar* (‘to bleed’)		867 (0.86)
**N/NPR**	*aspirina* (‘aspirin’)		107 (0.11)
**ADV**	*levemente* (‘mildly’)		40 (0.04)
**ADJ/ADV**	*in situ*		20 (0.02)

*Abbreviations: *M* Mean; *SD* Standard deviation; *CUI* Concept unique identifier; *N* Noun; *ADJ* Adjective; *NPR* Proper name: *V* Verb; *AFF* Affix; *ADV* Adverb; *ADJ/N* 'Adjective’ or ‘noun’ (depending on the context; *idem* for *ADJ*/*ADV* etc.)

**Fig. 6:**
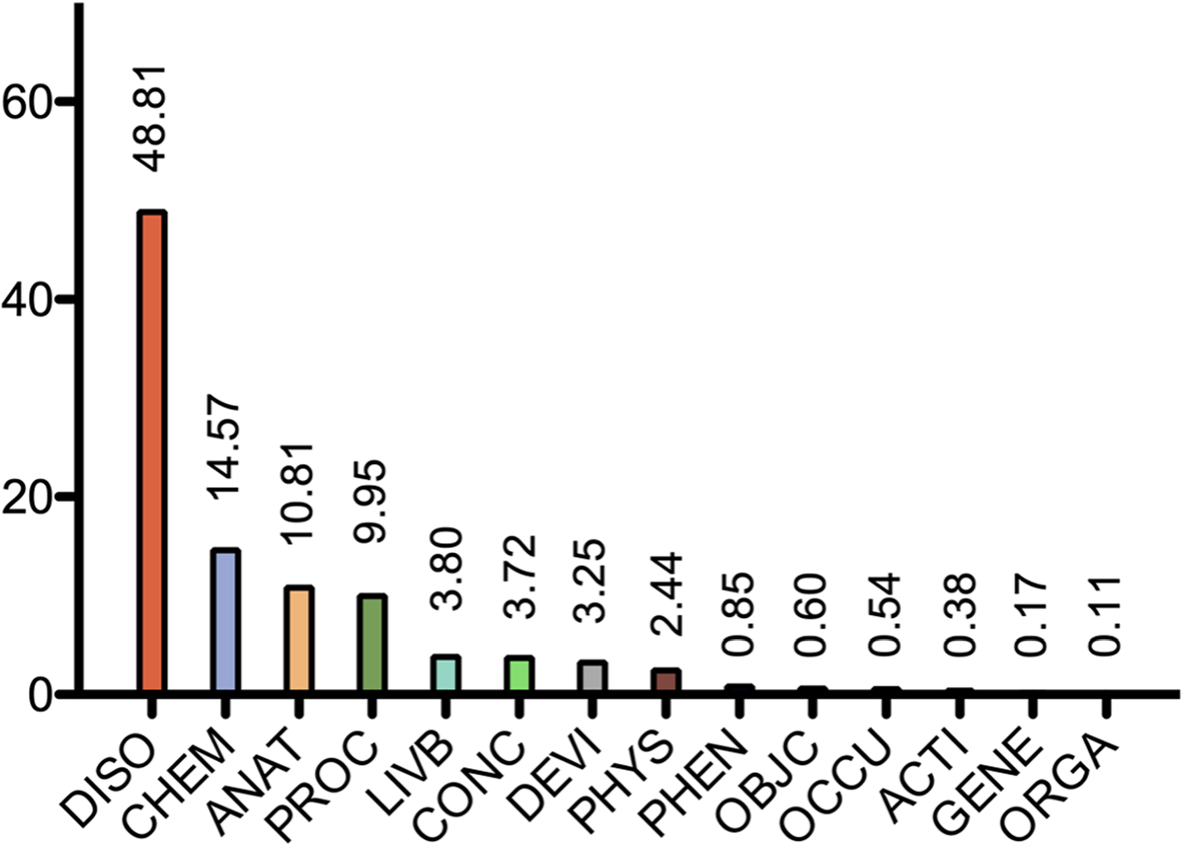
Distribution of semantic groups (%)

**Fig. 7:**
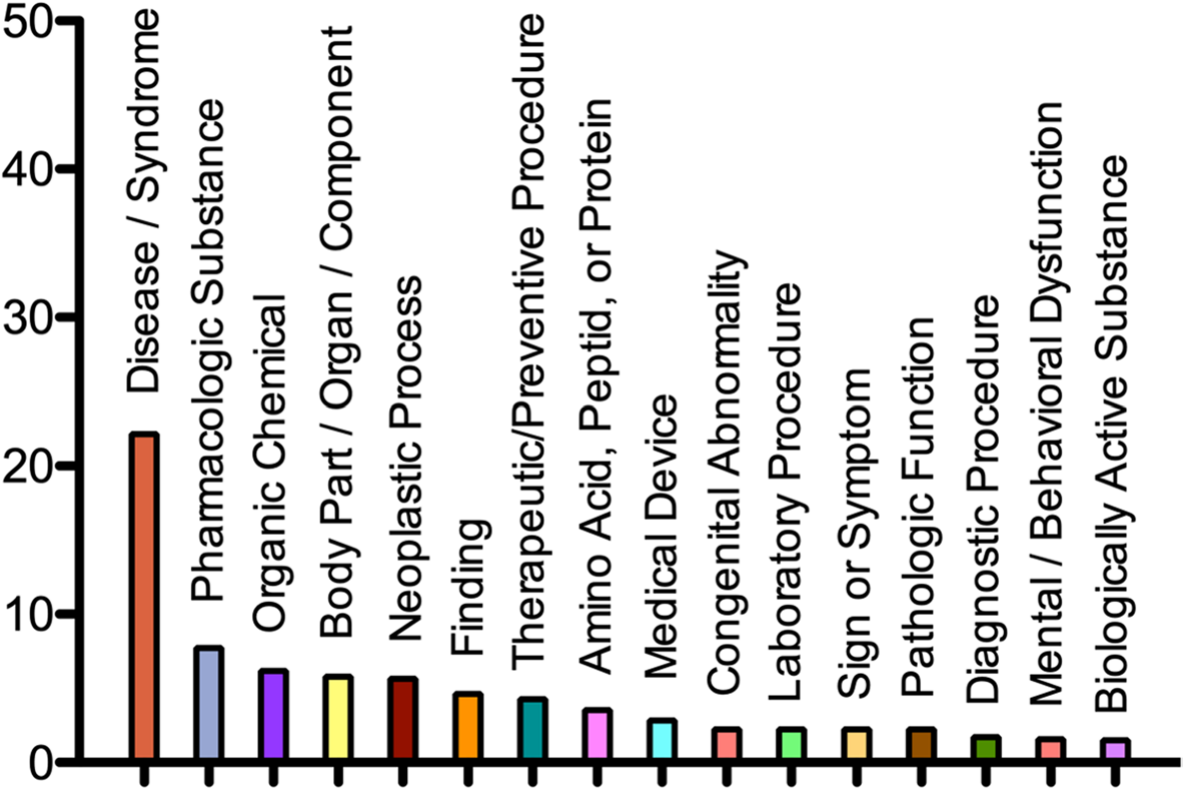
Most frequent semantic types (%)

Figures 6 and 7 depict, respectively, the distribution of UMLS semantic groups and the most frequent semantic types in MedLexSp. The current version gathers more than 25 000 terms of semantic type Disease_or_Syndrome, and over 8000 terms of type Pharmacologic_Substance. The types of corpora and thesauri used to extract terms may explain the fact that some groups are less frequent. For example, the GENE group is underrepresented; consequently, the current version of MedLexSp is not adequate for tasks in the field of Genomics. In contrast, the proportion of semantic types related to Neoplastic Process is larger than in the previous version of the lexicon. This is due to the fact that more terms from the CANTEMIST corpus have been included. Therefore, the cancer domain is represented better.

Tables 4 and 5 list, respectively, the part-of-speech categories considered and the morphological data categories, with examples.

**Table 4:** List of part-of-speech categories and linguistic data with examples

Part-of-speech (abbreviation)	Example
adjective (ADJ)	*severo* (‘severe’)
adjective_or_noun (ADJ/N)	*diabético* (‘diabetic’)
adjective_or_adverb (ADJ/ADV)	*in situ* (‘on site’)
adverb (ADV)	*levemente* (‘mildly’)
affix (AFF)	*reno-* (‘kidney’)
noun (N)	*hígado* (‘liver’)
noun_or_properNoun (N/NPR)	*aspirina* (‘aspirin’)
properNoun (NPR)	*apocard*$^{\circledR}$
verb (V)	*sangrar* (‘to bleed’)

**Table 5:** List of morphological data categories with examples

Attribute	Value	Example
grammaticalGender		
	commonGender	*leve* (‘mild’)
	masculine	*hombre* (‘man’)
	feminine	*embarazada* (‘pregnant’)
grammaticalNumber		
	singular	*pulmón* (‘lung’)
	plural	*pulmones* (‘lungs’)
	singular_and_plural	*diabetes*
person		
	firstPerson	*sudo* (‘I sweat’)
	secondPerson	*sudas* (‘you sweat’)
	thirdPerson	*suda* (‘he/she sweats’)
grammaticalTense		
	present	*tose* (‘he/she coughs’)
	imperfect	*tosían* (‘they coughed’)
	past	*tosió* (‘he/she coughed’)
	future	*toserá* (‘he/she will cough’)
	conditional	*tosería* (‘he/she would cough’)
	presentPerfect	*te has atragantado* (‘you have choked’)
verbFormMood		
	indicative	*tose* (‘he/she coughs’)
	subjunctive	*tosa* (‘he/she coughs’)
	imperative	*tose (tú)* (‘cough’)
	infinitive	*toser* (‘to cough’)
	gerund	*tosiendo* (‘coughing’)
	participle	*tosido* (‘coughed’)
VariantType		
	abbreviation	*Dr.* (‘doctor’)
	acronym	*SIDA* (‘AIDS’)

**Fig. 8:**
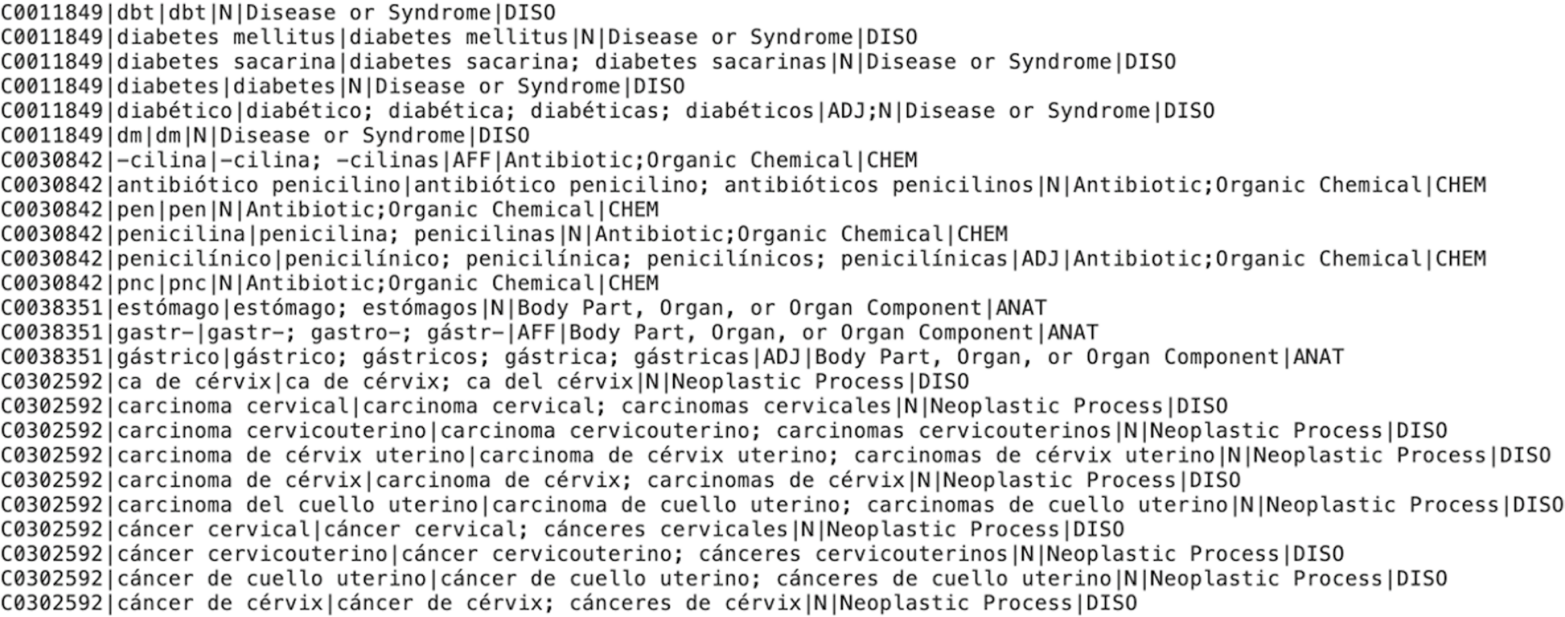
Sample of MedLexSp in delimiter separated values (dsv) format. Field 1 is the UMLS CUI of the entity; field 2, the lemma; field 3, the variant forms; field 4, the part-of-speech; field 5, the semantic types(s); and field 6, the semantic group

**Fig. 9:**
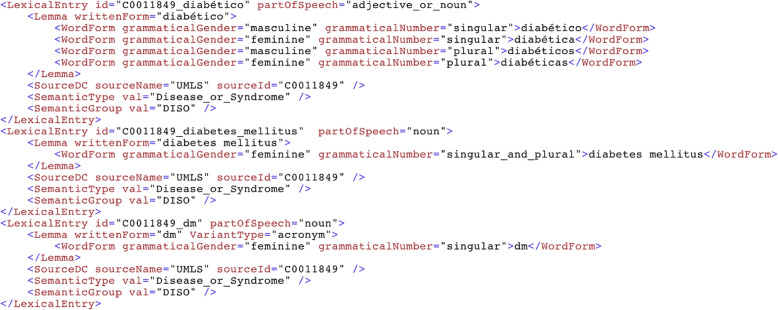
Sample of lexical entries in the Lexical Markup Framework

**Fig. 10:**
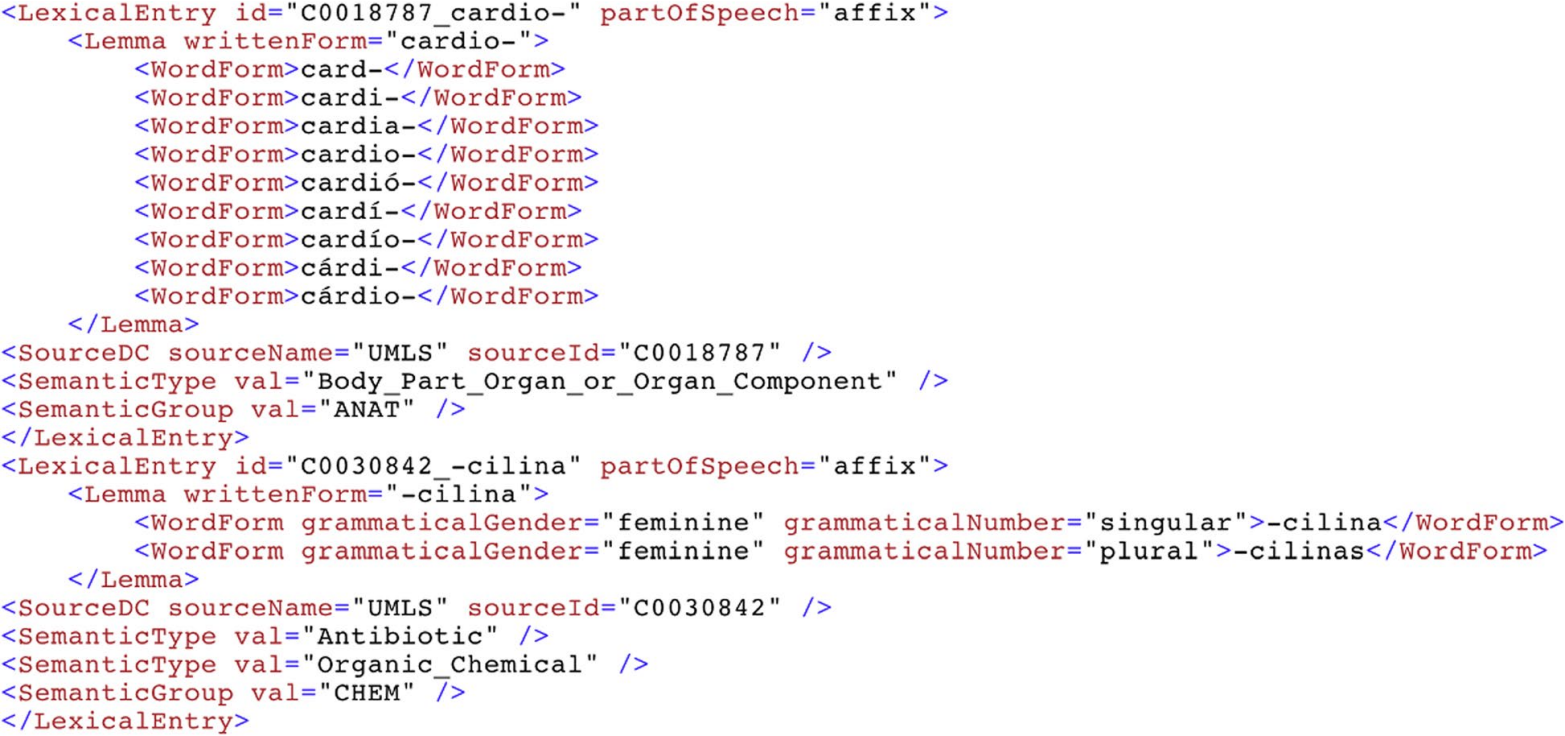
Sample of prefixes and suffixes in the Lexical Markup Framework

**Fig. 11:**
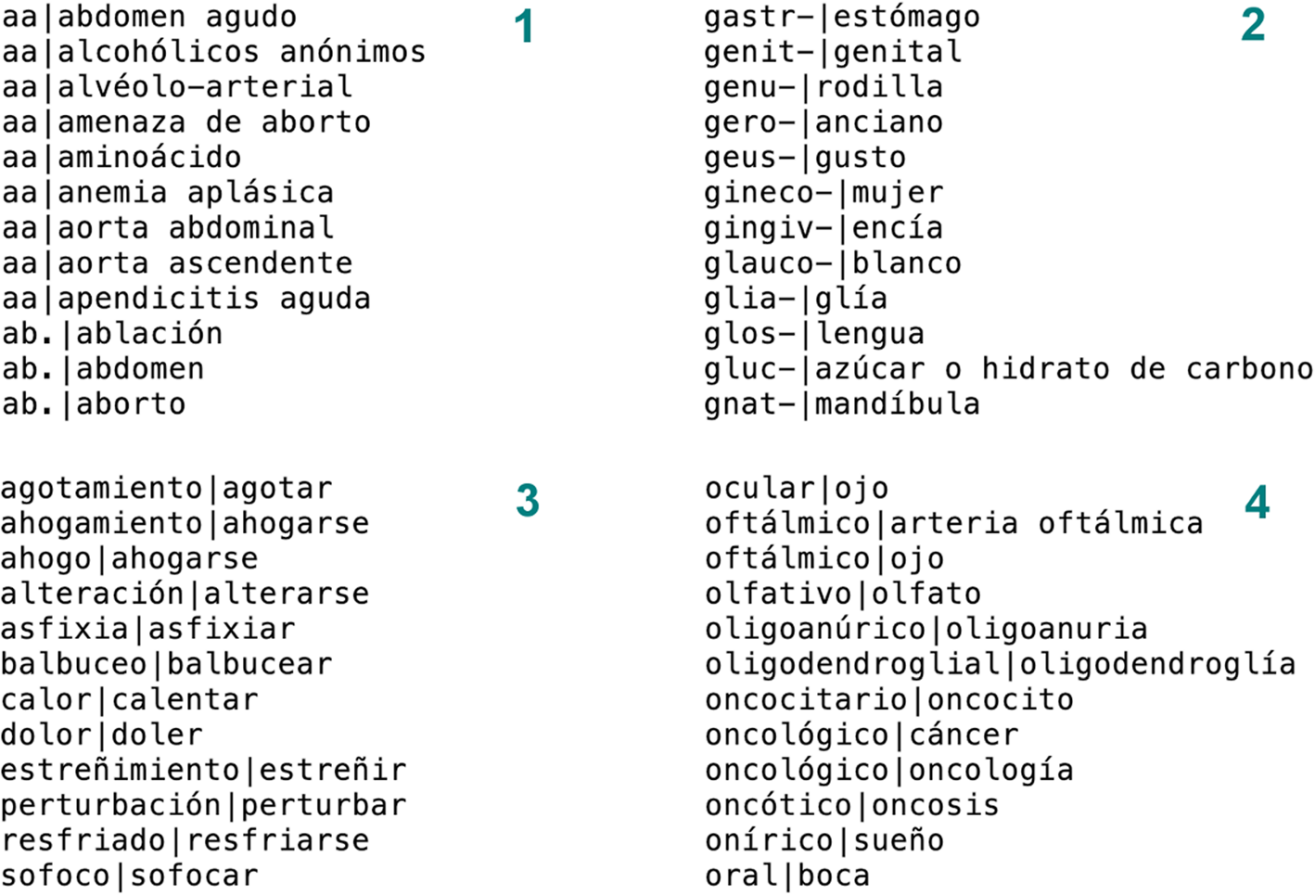
Sample of Lexical Record (LR) files: 1) abbreviations/acronyms and full forms; 2) affixes and their meanings; 3) deverbal nouns and verbs; and 4) nouns and adjectives derived from (or related to) nouns

The MedLexSp lexicon is distributed freely for research and educational purposes in several formats:

1. A delimiter-separated value file, which is similar to MRCONSO.RRF or MRSTY.RRF files in the UMLS Metathesaurus (but with less data fields) (Fig. 8).
2. An XML-encoded version using the Lexical Markup Framework (LMF), which includes the morphological data (number, gender, verb tense and person, and information about affix/abbreviation data). Figure 9 shows a sample of lexical entries for different term variants of the concept *diabetes*: as an adjective (*diabético*, ‘diabetic’), a noun (*diabetes mellitus*) and an acronym (*dm*). Figure 10 shows a sample of prefixes and suffixes.
3. A lemmatizer module for the Spacy and Stanza python libraries. The Spacy lemmatizer includes 106 396 new variant forms with regard to the default Spacy distribution for Spanish; in total, the updated lemmatizer gathers 564 725 variant forms. The Stanza lemmatizer gathers new 104 551 variant forms.
4. Lexical Record (LR) files with equivalences between affixes/roots and their meanings, between acronyms/abbreviations and full forms, between nouns and deverbal nouns, and between nouns and adjectives derived from nouns (Fig. 11).

These resources are distributed with a license agreement to be signed. The Spacy and Stanza lemmatizers are available for immediate download at the companion repository (see Availability of data and materials).

## Utility and discussion

### Use cases

As a first use case, MedLexSp was used to pre-annotate the Clinical Trials for Evidence-based Medicine in Spanish (CT-EBM-SP) corpus. This is a collection of 1200 texts (292 173 tokens): 500 abstracts of clinical trial studies published in journals from the SciELO repository [59] and 700 clinical trial announcements from EudraCT [60]. Three annotators with different backgrounds (a computational linguist, a medical lexicographer and a medical doctor) conducted the annotation. This corpus was used in a supervised context to train named entity recognition models with state-of-the-art deep neural models (SequenceLabeler [73], FLAIR [74] and BERT [4]). This resource is distributed in the community and full details are described in another article [23].

A second use case was part-of-speech (PoS) tagging and lemmatizing a collection of 100 texts from a public corpus that is PoS annotated and lemmatized. This corpus gathers clinical cases published in journal articles [75]. Particularly, we compare the results of using state-of-the-art python libraries (Spacy [25] and Stanza [26]) with and without MedLexSp. First, we selected the subset of 100 texts that was revised by two annotators—i.e. the sample with the expected highest annotation quality. These texts were the gold standard for the comparison; however, some PoS and lemmatization errors were found and fixed. The labels in this corpus were converted to the Universal Dependencies standard [76], which is used in Spacy and Stanza. Then, we part-of-speech tagged and lemmatized the 100 texts using Spacy and Stanza without MedLexSp, using the default lexicon and models. With Spacy, the medium size model (es_core_news_md) was used. We applied both libraries again using the MedLexSp lexicon. All experiments were conducted on a CPU of a laptop (not a GPU). Because MedLexSp gathers open (lexical) PoS categories, only these were compared: namely, adjectives, adverbs, nouns, proper nouns and verbs. For comparing the lemmatization, minor variations in lemmas were not considered errors (e.g. use of accent or not: *cardíaco* vs. *cardiaco*, ‘cardiac’). The evaluation metrics were standard precision, recall and F1 measures, computed with the ScikitLearn library [77].

### Results of the use cases

In the first use case, the pre-annotation of 1200 health texts helped the annotators label entities of four UMLS groups (ANAT, CHEM, DISO and PROC). A total of 56 343 entities were pre-annotated, and after the manual human revision, the number of annotations decreased to 46 699 entities (17.12% of the pre-annotated entities were removed). Per sub-corpus, the number of pre-annotated entities was 25 265 (journal abstracts) and 31 078 (clinical trial announcements). After manual revision, the number of entities decreased to 20 031 in journal abstracts (20.71% of the pre-annotations were removed) and to 26 668 in trial announcements (14.19% of the pre-annotations were removed). These pre-annotated entities were eliminated due to several reasons. First, wrong sense of polysemous entities: e.g. *vacuna* may be a verb expressing a therapeutic procedure (‘he/she vaccinates’) or a medical drug (‘vaccine’). Second, overlapping of general and specific entities (the less specific entity was removed): e.g. *dolor* (‘pain’) and *dolor de cabeza* (‘headache’). Lastly, crossing entities (with a span that overlaps in some words) were corrected to independent entities: e.g. in *administración de vacunas vivas* (‘administration of live vaccines’) there is an overlap in *vacunas* of two entities (*administración de vacunas* and *vacunas vivas*), which was corrected. The full annotation process lasted over seven months. The inter-annotator agreement (IAA) scores were high: an average F1 of 85.65% (±4.79) in a strict match setting (i.e. when annotators agreed both in the scope and class of the annotated entity), and an average F1 of 93.94% (±3.31) (relaxed match). Training and testing with this corpus, the named entity recognition models achieved results with an average F-measure that ranged from 80.28% (±00.99) to 86.74% (±00.19) in the test set.

In the second use case, comparing the PoS-tagging and lemmatization with and without MedLexSp showed the benefits of using a domain specific lexicon. Table 6 reports the average precision (P), recall (R) and F1-measure when using Spacy and Stanza alone, or combined with MedLexSp (standard deviation values are shown in brackets). For both tasks, using MedLexSp yielded higher scores. Stanza with MedLexSp achieved the highest scores (marked in bold): average F1 = 94% for PoS tagging, and average F1=96% for lemmatization; it especially improved precision values. Remember that these results correspond only to lexical categories (adjectives, adverbs, nouns, proper nouns and verbs). The processing times were slightly faster using Spacy in a CPU: 100 texts in 10 minutes with MedLexSp, and in 9’ 41” without it. Stanza’s processing times were 12’ 22” and 12’ 1”, respectively with and without MedLexSp.

**Table 6:** Part-of-speech tagging and lemmatization with/without MedLexSp (MLS)

	Part-of-speech tagging			Lemmatization		
	P	R	F1	P	R	F1
**Spacy**	91.3	87.0	88.7	93.6	92.9	93.1
	(±3.7)	(±5.1)	(±4.4)	(±2.3)	(±2.4)	(±2.4)
**Spacy**	93.3	91.9	92.3	95.4	95.1	95.1
**+ MLS**	(±3.0)	(±2.9)	(±2.9)	(±2.1)	(±2.1)	(±2.1)
**Stanza**	94.7	**95.5**	92.2	95.4	95.0	95.1
	(±2.3)	**(±4.2)**	(±3.6)	(±2.0)	(±2.0)	(±2.0)
**Stanza**	**94.9**	**93.7**	**94.0**	**96.3**	**96.0**	**96.0**
**+ MLS**	**(±2.2)**	**(±2.7)**	**(±2.6)**	**(±1.8)**	**(±1.8)**	**(±1.8)**

We conducted a shallow analysis of PoS errors. Regarding the performance per category, Table 7 reports the average F1 score and standard deviation in brackets. In adverbs, the highest scores were achieved with MedLexSp (F1 = 94.05% with Spacy and F1 = 95.55% with Stanza), but the difference without MedLexSp was not large. Nouns had the highest scores when using MedLexSp (average F1 = 95.59% with Spacy and F1 = 96.75% with Stanza). Adjectives achieved an average F1 measure of 90.69% using Stanza without MedLexSp, which improved to 91.06% (Spacy) and to 92.28% (Stanza) when using MedLexSp. The performance of verbs also improved when using MedLexSp (particularly by rising the recall): the average F1 measure raised from 87.11% to 88.64 % with Spacy, and from an average F1 of 88.71% to 89.29% with Stanza. The main source of verb errors were related to past participle forms that can be considered adjectives: e.g. *ulcerada*, ‘ulcerated’, adjective (lemma: *ulcerado*) or verb (lemma: *ulcerar*). Proper nouns had the lowest scores; using MedLexSp helped, but the performance was not high. Many of these errors appeared in uppercase words: Spacy or Stanza always tagged them as proper nouns, although they can bear a different category (they may appear in uppercase at the beginning of the sentence). Other errors in proper nouns are related to eponyms (e.g. *Doppler*) and medical drugs (e.g. *ertapenem*). MedLexSp improved the performance in these cases, except in terms without a CUI (e.g. brand names such as *Trigon Depot*). Acronyms were another source of errors: e.g. Stanza and Spacy tagged *UCI* (‘intensive care unit’) or *VIH* (‘human immunodeficiency virus’) as proper noun, but MedLexSp tagged them correctly as nouns. Other errors affected medical adjectives that were tagged as nouns: e.g. Stanza mislabeled *úrico* (‘uric’) and Spacy miscategorized *digestiva* (‘digestive’), but both are adjectives (MedLexSp assigned the correct category). Finally, some errors could not be solved even with the lexicon. As said, most occurred in past participle forms, which were often tagged as adjectives. Also, many PoS errors affected words that may be either adjective or noun; frequently, these refer to the pharmacological action or the drug class: e.g. *antiemético*, adjective (‘antiemetic’) or noun (‘antiemetic agent’).

**Table 7:** Performance of PoS tagging per category with/without MedLexSp

	P	R	F1	P	R	F1
	**Spacy**			**Spacy + MedLexSp**		
**ADJ**	83.64	91.10	87.04	88.03	94.58	91.06
	(±7.21)	(±5.62)	(±5.33)	(±6.08)	(±4.96)	(±4.64)
**ADV**	96.13	92.76	93.36	98.34	91.84	94.05
	(±8.95)	(±15.22)	(±11.20)	(±5.01)	(±15.49)	(±10.50)
**NOUN**	95.29	88.34	91.62	95.84	95.47	95.59
	(±3.03)	(±6.95)	(±4.49)	(±2.75)	(±3.15)	(±2.19)
**PROPN**	10.48	24.16	13.11	18.75	25.30	19.49
	(±18.50)	(±34.04)	(±20.58)	(±26.68)	(±34.37)	(±25.62)
**VERB**	95.74	80.52	87.11	97.57	81.76	88.64
	(±5.61)	(±8.56)	(±5.82)	(±3.96)	(±8.96)	(±5.72)
	**Stanza**			**Stanza + MedLexSp**		
**ADJ**	87.06	94.99	90.69	89.05	95.83	**92.28**
	(±5.97)	(±3.86)	(±3.74)	(±5.66)	(±3.97)	**(±3.85)**
**ADV**	99.38	93.31	95.40	99.88	92.97	**95.55**
	(±5.13)	(±14.85)	(±10.40)	(±1.20)	(±14.81)	**(±9.97)**
**NOUN**	97.94	91.67	94.52	96.77	96.83	**96.75**
	(±1.93)	(±6.75)	(±4.11)	(±2.30)	(±2.92)	**(±2.11)**
**PROPN**	36.08	78.45	45.49	49.72	52.47	**47.92**
	(±30.70)	(±40.39)	(±32.79)	(±39.28)	(±39.26)	**(±35.66)**
**VERB**	98.87	80.88	88.71	98.77	82.05	**89.29**
	(±2.77)	(±8.96)	(±5.74)	(±2.75)	(±8.69)	**(±5.29)**

*Abbreviations: ADJ: adjective; ADV: adverb; PROPN: proper noun

Regarding lemmatization, we found errors using Stanza or Spacy that were lemmatized correctly using MedLexSp (Table 8). These generally occur when the lemma ends with *-s* (which normally corresponds to the plural form in Spanish) or with *-a* (which normally expresses the feminine gender). Other errors were due to incorrect PoS tagging; they occurred either when using or not MedLexSp, and varied across texts (depending on how each linguistic context affected the PoS prediction). For example, *alta*, as a feminine noun, refers to ‘discharge’, but was often tagged as an adjective (the feminine of *alto*, ‘tall’). Another example is *evidencia*, which can be a noun (‘evidence’, lemma: *evidencia*) or a verb (third person singular of the present tense, lemma: *evidenciar*, ‘to evidence’). The last type of errors were those that were not solved even when using MedLexSp. There were differences in lemmas of numeral adjectives: e.g. *décimo* (‘tenth’) was lemmatized as *10* in the gold standard. Neither Stanza nor Spacy lemmatized them correctly. However, these were not medical terms, and the impact on lemmatization performance was not critical. Other errors were due to segmentation or spelling mistakes in the corpus: e.g. *realizron, ‘made’ (which was correctly lemmatized as *realizar* in the gold standard) or *ne-froureterectomía, ‘nephroureterectomy’ (lemmatized as *nefroureterectomía* in the gold standard). Compound words, which are very productive in the medical domain, were another source of errors. Stanza lemmatized *vesico-prostática* (‘vesical-prostatic’) as *vesico-pro, which does not exist in Spanish. Spacy did not produce these types of errors. For example, Spacy lemmatized correctly *uretra-neovejiga* (‘urethra-neobladder’), the form being the same as the lemma. The lemmatization methods of Spacy and Stanza explain these differences. Spacy uses a lemmatization module to map forms to lemmas; in the case of out-of-vocabulary (OOVs) words, the heuristic is using the unknown form for the lemma. Stanza uses the LemmaProcessor, which combines a dictionary-based and a neural seq2seq lemmatizer (applied by default). In the case of out-of-vocabulary (OOVs) words, Stanza caused lemmatization errors by creating non-existing words in Spanish. For the previous example of OOV word items, Stanza lemmatized it as *uretra-neovejigigo. Note that we also tested Stanza with the method of using the form as the lemma for OOV items (lemma_use_identity = True). However, the results were worse (average F1=84.8±3.0), mostly due to lemmatization errors of conjugated verb forms.

**Table 8:** Examples of lemmatization errors (asterisks mark non-existing words)

Word form	Spacy	Stanza	+MedLexSp
*corticoides*	*corticoid	*corticoid	*corticoide*
(‘corticosteroids’)			
*evidencia* (‘evidence’)	*evidenciar*	*evidenciar*	*evidencia*
*hematíes*	*hematí	*hematí	*hematíe*
(‘red blood cells’)			
*hemodiálisis*	*hemodialisi	*hemodiálisis*	*hemodiálisis*
(‘hemodialysis’)			
*inmunohistoquímica*	*inmunohistoquímica*	*inmunohistomico	*inmunohistoquímico*
(‘immunohistochemical’)			
*páncreas* (‘pancreas’)	*páncreas*	*páncrea	*páncreas*
*piuria* (‘pyuria’)	*piurio	*piuria*	*piuria*

### Discussion

Medical lexicons enable actionable processing of texts in natural language, and are more powerful than gazetteers, especially for part-of-speech (PoS) tagging and lemmatization. The main issue when curating a domain-specific lexicon lies in achieving enough coverage [37]: What types of words should be included as medical terms? And more importantly: To what extent the most important terms and semantic classes are represented? We think the methods used to create this lexicon have addressed these challenges. By collecting terms from corpora used in shared tasks (e.g. PharmaCoNER or CANTEMIST) and from patient-oriented resources (e.g. NCI or MedlinePlus), MedLexSp gathers *real-usage* terms. In addition, by curating terms from terminologies, taxonomies and ontologies (e.g. ICD-10, MeSH or SNOMED-CT), MedLexSp guarantees a high coverage of standard medical thesauri and makes it possible the interoperability across thesauri in concept normalization tasks. The aspect of exhaustiveness was tackled by generating word order variants, taking into account morphological term variants (verbs and deverbal nouns, adjectives derived from nouns, and affixes and their meanings), and collecting the full forms of acronyms and abbreviations. Lastly, the problem of neologisms—i.e. new medical concepts giving rise to new terms—was faced when the COVID-19 pandemic rose up. We experimented with word embeddings and seed words to gather new variants of terms that are semantically close in the vector space. Interestingly, smaller word embeddings, but trained with texts related to the topic, yielded better results than embeddings trained in larger collections. In an attempt to demonstrate the maturity of MedLexSp, we reported two use cases. In particular, the evaluation conducted on PoS tagging and lemmatization (using MedLexSp or not with state-of-the-art python libraries) showed that this lexicon raised the F1 scores for both tasks.

With regard to the first use case, using MedLexSp for pre-annotation allowed the annotators to easily detect or confirm the entities to be annotated. The pre-annotation could also explain the high inter-annotator agreement (IAA) scores obtained. However, no comparison was made in an annotation setting without pre-annotation. Therefore, the effect on the IAA values remains to be confirmed. Moreover, a disadvantage of pre-annotation was causing some false positives or mismatches that annotators had to fix or delete during the manual revision. There is a trade-off between speeding up the annotation task and causing redundant or noisy annotations. However, the count of deleted pre-annotations during revision was not large (17.10%): overall, our experience with the lexicon-based pre-annotation method was positive.

The evaluation of the second use case—POS-tagging and lemmatization—showed the advantages of feeding general purpose tools (in our example, Spacy and Stanza) with a dedicated lexicon to improve the scores. Both tools increased the F1 scores in combination with MedLexSp. Stanza achieved the highest F1 measures in both PoS tagging and lemmatization. However, we found critical errors: e.g. *síndrome* (‘syndrome’) was lemmatized as *sendrar (a non-existing verb in Spanish) plus *me* (first person singular pronoun). Besides, for OOV words, Stanza created non-existing lemmas, whereas Spacy took the same form (which might be the lemma in some cases). Altogether, the error analysis showed that many PoS errors depend on how the linguistic context affected the model’s prediction for ambiguous words (e.g. *fumador*, ‘smoker’, adjective or noun; or *irradiado*, ‘irradiated’, past participle and verb). Solving these errors would require training a specific part-of-speech model with a tagged corpus, which is out of the scope of this work. Anyhow, these are errors that are not expected to have a severe impact on any task. Lastly, several errors affected terms (especially, proper names) of brand names, eponyms or acronyms that were not included in the lexicon (because they lack a CUI). Also, spelling and tokenization mistakes in the source text affected the performance. Nonetheless, all those types of errors occurred with low frequency.

As exposed above, a limitation of this version of MedLexSp is the fact that it does not contain terms without UMLS CUIs. For example, some brand names (e.g. *Progevera*$^{\circledR }$), spatial adjectives or qualifiers (e.g. *abdominogenital*). A solution to overcome this limitation would be assigning new non-UMLS identifiers to missing medical terms in a future version. However, to avoid including noisy terms or variants that are not widely generalized, new terms should be included only if registered in several quality resources or databases created by health professionals or lexicographers (e.g. PubChem [78] or *Diccionario de Términos Médicos* [17]). Moreover, we did not consider other terminology sources, or only included very few terms from them. For example, MedLexSp only has 106 term entries from the National Center for Biotechnology Information (NCBI) taxonomy [79], which is a curated nomenclature of all the organisms in genetic databases. Besides, the uninterrupted creation of neologisms and medical concepts makes it necessary a continuous update. All these facts cause a lack of complete exhaustiveness and explain the limitations of lexicons for NLP. Nonetheless, even if a more comprehensive lexicon is created, each task will demand specific criteria to adapt the lexicon by filtering the most adequate term types. For example, in a cancer-related task, terms related to UMLS semantic types such as Neoplastic Process need to be used, but other semantic groups may cause noise. Previous works in concept normalization have brought up this issue [80].

Another limitation is the lack of syntactic information about subcategorization frames or syntactic behavior. This type of information is included in resources such as the Biolexicon [38]. MedLexSp neither encodes semantic relations between term entries: it is not a medical ontology at the current stage. The UMLS Semantic Network includes is_a relations between concepts (e.g. *hypertension*is_a*hypertension*) and also gathers relationships available in sources such as SNOMED-CT. Enriching MedLexSp with semantic relations would provide an ontology resource for text mining or information extraction at a higher level. Future directions are enriching the lexicon with more resources, and widening the coverage of terms from American Spanish. The next version should contain the *Pan-Hispanic Dictionary of Medical Terms*, which is currently under development by the Spanish National Academy of Medicine; and also include the equivalents of the Spanish *Nomenclator* for drug names in Spanish America. The next planned step is collecting consumer health terms and laymen variants (e.g. *amigdalectomía*, ‘tonsillectomy’ $\leftrightarrow$*operación de anginas*, ‘tonsils surgery’), also mapped to CUIs.

Despite these limitations, MedLexSp can contribute to concept normalization through a established standard (UMLS) and paves the way towards generating concept embeddings to be used in medical informatics tasks [81, 82]. In addition, the linguistic information included in this resource would allow natural language generation systems to improve the grammar correctness of the generated utterances in the health domain.

## Conclusion

This work has described the stable version of the Medical Lexicon for Spanish (MedLexSp), an unified medical vocabulary for natural language processing. Namely, we have reported the latest contributions: 1) Gathering new term lemmas and variant forms from the *Dictionary of Medical Terms* from the Spanish Royal Academy of Medicine [17]; 2) Collecting corpus-based terms documented in MedlinePlus and domain annotated corpora, in particular from recent shared tasks (PharmaCoNER, CODIESP and CANTEMIST) and domain text resources (CWLC and CT-EBM-SP corpora); 3) Enriching the linguistic information of each term with its part-of-speech class and morphological data (e.g. gender, number, and tense, person and mood in the case of verbs); 4) Testing an approach to collect new terms related to the COVID-19 pandemic by applying a similarity measure and word embeddings trained on a corpus about this topic; and 5) Presenting two use cases: using the lexicon to pre-annotate a corpus of 1200 health texts, and part-of-speech (PoS) tagging and lemmatizing 100 texts related to clinical cases. Comparing the performance with and without the lexicon showed an increase of PoS and lemmatization scores using MedLexSp.

The strengths of this lexicon have been discussed. Namely, the broad coverage of medical vocabulary, ensured by the terms extracted from domain corpus and resources used in recent BioNLP challenges, together with standard thesauri, classifications and ontologies (ATC, ICD-10, MedDRA, MeSH, NCI, OMIM or SNOMED-CT). MedLexSp is distributed in several formats: a delimiter-separated value file; an XML file modeled with the Lexical Markup Framework; a lemmatizer for Spacy and Stanza python libraries; and complementary Lexical Record (LR) with equivalences between affixes/roots and their meanings, full forms and acronyms/abbreviations, nouns and deverbal nouns or adjectives derived from nouns. These different formats allow a flexible and actionable use of this resource for natural language processing tasks such as part-of-speech tagging, lemmatization, concept normalization or natural language generation.

The limitations of MedLexSp have been pinpointed. One is the lack of a comprehensive exhaustiveness of terms, because words not registered in the UMLS were not included. Another weakness is the fact that some semantic types are under-represented—namely, genomic terms and gene names, which limits the use of MedLexSp for such type of content. Future work will involve enlarging this resource with more sources, varieties of Spanish and with consumer health terms, and enriching the linguistic and domain information available. The Spacy and Stanza lemmatizer modules, and the code and data for the word-embedding experiments are available at the companion repository.

## Data Availability

The MedLexSp lexicon is available for research and educational purposes at the Digital.CSIC repository, https://digital.csic.es/handle/10261/270429 (https://doi.org/10.20350/digitalCSIC/14656). Please, contact for a license. Terms and lexical information from the *Dictionary of Medical Terms* were obtained via a signed agreement with the Spanish Royal Academy of Medicine (RANME). Some thesauri included in MedLexSp were obtained through a distribution and usage agreement from the corresponding institutions who develop them. In addition, some material in the UMLS Metathesaurus is from copyrighted sources of the respective copyright holders. Users of the UMLS Metathesaurus are solely responsible for compliance with any copyright, patent or trademark restrictions and are referred to the copyright, patent or trademark notices appearing in the original sources, all of which are hereby incorporated by reference. The version of MedLexSp freely available for research does not include terms nor coding data from terminological sources with copyright rights. We acknowledge the intellectual property rights of the institutions who develop the sources from which we extracted subsets of terms to compile the lexicon, and we are very thankful for having given permission (or provided a license to reuse their data) to distribute their resources. The Spacy and Stanza lemmatizers, the word embeddings and the source code used to extract new terms about the COVID-19 are available at the following repository: https://github.com/lcampillos/MedLexSp

## Abbreviations

ATC: Anatomical Therapeutic Chemical Classification
CANTEMIST: CANcer TExt Mining Shared Task
CODIESP: Clinical Case Coding in Spanish
CUI: Concept Unique Identifier
CWLC: Chilean Waiting List Corpus
CT-EBM-SP: Clinical Trials for Evidence-based Medicine in Spanish
DSM-5: Diagnostic and Statistical Manual of Mental Disorders, 5th edition
DTM: *Diccionario de términos médicos* (‘Spanish dictionary of medical terms’)
ICD-10: International Classification of Diseases vs. 10
ICPC: International Classification of Primary Care
LMF: Lexical Markup Framework
M: mean
MedDRA: Medical Dictionary for Regulatory Activities Terminology
MeSH: Medical Subject Headings
NCI: National Cancer Institute
NLP: Natural language processing
OMIM: Online Mendelian Inheritance in Man
PoS: Part-of-speech
SD: Standard deviation
SDEdb: Spanish Drug Effect database
SNOMED-CT: Systematized Nomenclature of Medicine - Clinical Terms
SPC: Summary of Product Characteristics
t-SNE: t-Distributed Stochastic Neighbor Embedding
UMLS: Unified Medical Language System$^{\circledR }$
WHO: World Health Organization
